# Inhibitory modulation of cytochrome *c* oxidase activity with specific near-infrared light wavelengths attenuates brain ischemia/reperfusion injury

**DOI:** 10.1038/s41598-018-21869-x

**Published:** 2018-02-22

**Authors:** Thomas H. Sanderson, Joseph M. Wider, Icksoo Lee, Christian A. Reynolds, Jenney Liu, Bradley Lepore, Reneé Tousignant, Melissa J. Bukowski, Hollie Johnston, Alemu Fite, Sarita Raghunayakula, John Kamholz, Lawrence I. Grossman, Karin Przyklenk, Maik Hüttemann

**Affiliations:** 1 Department of Emergency Medicine, Wayne State University School of Medicine, Detroit, MI 48201 USA; 2 Department of Emergency Medicine, University of Michigan Medical School, Ann Arbor, MI 48109 USA; 3 Department of Molecular and Integrative Physiology, University of Michigan Medical School, Ann Arbor, MI 48109 USA; 4 Cardiovascular Research Institute, Wayne State University School of Medicine, Detroit, MI 48201 USA; 5 Department of Physiology, Wayne State University School of Medicine, Detroit, MI 48201 USA; 6 Center for Molecular Medicine and Genetics, Wayne State University School of Medicine, Detroit, MI 48201 USA; 7 College of Medicine, Dankook University, Cheonan-si, Chungcheongnam-do 31116 Republic of Korea

## Abstract

The interaction of light with biological tissue has been successfully utilized for multiple therapeutic purposes. Previous studies have suggested that near infrared light (NIR) enhances the activity of mitochondria by increasing cytochrome *c* oxidase (COX) activity, which we confirmed for 810 nm NIR. In contrast, scanning the NIR spectrum between 700 nm and 1000 nm revealed two NIR wavelengths (750 nm and 950 nm) that reduced the activity of isolated COX. COX-inhibitory wavelengths reduced mitochondrial respiration, reduced the mitochondrial membrane potential (ΔΨ_m_), attenuated mitochondrial superoxide production, and attenuated neuronal death following oxygen glucose deprivation, whereas NIR that activates COX provided no benefit. We evaluated COX-inhibitory NIR as a potential therapy for cerebral reperfusion injury using a rat model of global brain ischemia. Untreated animals demonstrated an 86% loss of neurons in the CA1 hippocampus post-reperfusion whereas inhibitory NIR groups were robustly protected, with neuronal loss ranging from 11% to 35%. Moreover, neurologic function, assessed by radial arm maze performance, was preserved at control levels in rats treated with a combination of both COX-inhibitory NIR wavelengths. Taken together, our data suggest that COX-inhibitory NIR may be a viable non-pharmacologic and noninvasive therapy for the treatment of cerebral reperfusion injury.

## Introduction

Cardiac arrest impairs systemic perfusion and renders the brain and other organs ischemic. The brain is highly reliant on a constant supply of oxygen and nutrients and thus is particularly sensitive to the resultant deficit in blood flow. In the absence of rapid resuscitation and restoration of blood flow, ischemia leads to severe neurologic damage. While resuscitation is required to preserve neurologic function, it also initiates reperfusion injury, which plays a major role in the compromised recovery of resuscitated patients, and is thought to contribute significantly to the high incidence of morbidity and mortality associated with cardiac arrest. Despite advances in cardiopulmonary resuscitation, neurological function – a primary metric for clinical outcome – remains poor following cardiac arrest and resuscitation.

Excessive reactive oxygen species (ROS) generation from the mitochondrial electron transport chain (ETC) was proposed as a principal mechanism of neuronal cell death during reperfusion^1^. The ETC is a series of four protein complexes that utilize energy from electron donors to pump protons across the inner mitochondrial membrane to establish the mitochondrial membrane potential (ΔΨ_m_), which powers ATP synthesis. The activity of the ETC complexes is controlled by ΔΨ_m_ and, in particular, by post-translational modifications to maintain optimal intermediate ΔΨ_m_ values between 120–140 mV, which allow efficient energy production with minimal ROS generation (reviewed in^2,3^). Importantly, as ΔΨ_m_ hyperpolarizes beyond the physiological range (>140 mV), ROS production at ETC complexes I and III increases exponentially^4–6^.

Ischemia causes energy depletion and excessive calcium release^7^, which is a major signal for mitochondrial activation and global dephosphorylation of mitochondrial proteins^8,9^. We and others have proposed that calcium-activated dephosphorylations promote hyperactivation of the ETC complexes, resulting in prolonged hyperpolarization of ΔΨ_m_ and generation of ROS during reperfusion when oxygen, the terminal ETC substrate, reenters the ischemic tissue^3,10^. Accordingly, if ROS generation could be reduced during reperfusion, the consequences of ischemia/reperfusion (I/R) injury could be substantially ameliorated. In fact, reducing ROS damage is the goal of many proposed therapies for I/R injury^11^. However, pharmacological approaches to address ROS damage suffer from a fundamental limitation: effective drug concentrations in at-risk tissue rely on delivery via blood flow that is only established upon reperfusion.

Our aim was to provide a novel means of protecting the brain from I/R injury by developing therapeutic near infrared light (NIR) as a potential neuroprotective strategy for preserving neurologic function following cardiac arrest and resuscitation. As NIR delivery is not dependent on cerebral perfusion, such a non-invasive therapeutic approach has the potential to circumvent this barrier to early intervention for reperfusion.

It is generally agreed that NIR stimulates mitochondria through its interaction with cytochrome *c* oxidase (COX)^12,13^. COX contains several chromophores, including two copper centers that are involved in enzyme catalysis. Copper broadly absorbs NIR in the range of 700–1000 nm, and the copper centers in COX have been suggested to function as the primary photoacceptors for NIR^14–16^. Indeed, although the precise mechanisms by which NIR modulates aerobic metabolism are not fully understood, COX (the proposed rate-limiting ETC complex) is an attractive candidate for therapeutic intervention because it indirectly controls ΔΨ_m_ and thus ROS production (reviewed in^2^).

In the current study, we have discovered two NIR wavelengths that partially inhibit COX activity *in vitro* and investigated their ability to interrupt the mechanisms responsible for neuronal cell death caused by reperfusion injury. Most notably, we report that suppression of COX activity with 750 nm and/or 950 nm NIR reduces mitochondrial respiratory rate and ΔΨ_m_, prevents ROS generation in stress states including during reperfusion, and provides robust neuroprotection in an animal model of global brain I/R injury.

## Results

### Identification of COX-inhibitory NIR

**Figure 1:**
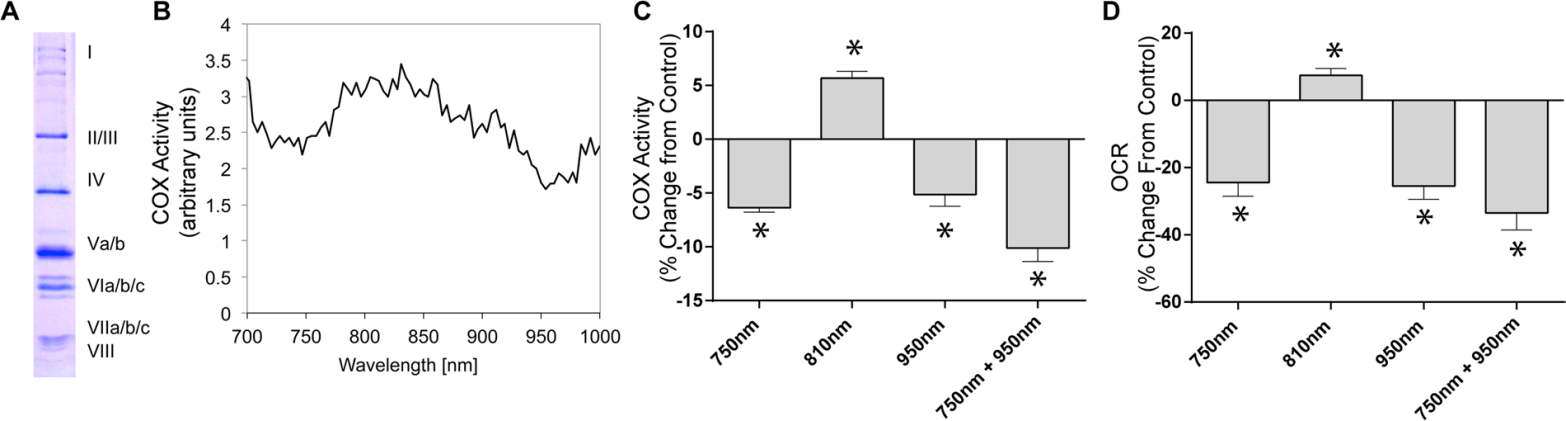
NIR modulates COX activity and mitochondrial oxygen consumption rate. (**A**) Isolated regulatory-competent bovine COX separated into its subunits on a high-resolution urea/SDS-PAGE Coomassie-stained gel. Subunits are indicated in roman numerals. (**B**) Representative scan of wavelength-dependent COX activity identifying the 750 nm and 950 nm wavelength rages as inhibitory regions. (**C**) Effect of NIR emitted by LED diodes confirms that 750 nm and 950 nm NIR inhibit COX *in vitro* while 810 nm NIR activates the enzyme. Data were obtained over a 3-min interval of irradiation and normalized to non-irradiated samples (n ≥ 4; *p < 0.05). (**D**) NIR irradiation modulates oxygen consumption rate (OCR) in a wavelength specific manner. 750 nm and 950 nm NIR reduce OCR below the basal respiration rate and 810 nm NIR increase mitochondrial OCR (n ≥ 4; *p < 0.05).

We systematically screened the NIR electromagnetic spectrum in the “therapeutic window of opportunity” of 700 nm to 1000 nm, where NIR absorptions by water and blood are minimal, allowing deep tissue penetration of the NIR for possible medical applications. We integrated a light-protected oxygen electrode chamber into a double beam spectrophotometer (see Materials and Methods), which contained regulatory-competent bovine COX purified under conditions preserving the physiological regulatory properties of the enzyme, such as posttranslational modifications (Fig. 1A). While the NIR frequencies were scanned, COX activity was measured simultaneously. In contrast to previous studies concluding that NIR consistently activates COX^12,17–22^, we identified two novel wavelength ranges (750 nm and 950 nm) that inhibit COX activity (Fig. 1B).

LEDs that cover the NIR range in 5–10 nm increments are commercially available and may be useful for medical applications. We obtained 750 nm and 950 nm low power diodes and we also included 810 nm diodes as a control wavelength since it has the opposite effect on COX. Since the diodes have a broader bandwidth (about ±30 nm) compared to the 8 nm bandwidth used in our scan, we first confirmed the effect of the LED emitted NIR on purified COX *in vitro* by fitting them into the windows of the light-protected oxygen electrode chamber. We observed inhibition of COX by 750 and 950 nm NIR in the range of 5–6%, whereas 810 nm NIR activated the enzyme to a similar extent (Fig. 1C). Interestingly, simultaneous irradiation with 750 and 950 nm NIR resulted in an additive effect, producing a 10% reduction in COX-specific O_2_ consumption rate.

### NIR modulates mitochondrial respiration

To establish the effect of NIR on mitochondria, we next isolated intact mitochondria. Mitochondrial respiration, shown as a % of basal respiratory rate, was reduced by NIR irradiation at 750 and 950 nm in the range of 24–25%, whereas 810 nm NIR increased mitochondrial oxygen consumption (Fig. 1D). Simultaneous irradiation with 750 and 950 nm NIR reduced mitochondrial respiration by 34%. Interestingly, the inhibitory effect of both 750 nm and 950 nm IRL was more pronounced in isolated mitochondria compared to purified COX. It should be noted that the percent change seen in mitochondrial respiration and isolated COX activity cannot be directly compared because the enzymatic environment is fundamentally different, including the presence of supercomplexes in intact mitochondria and, more importantly, the mitochondrial membrane potential, which itself is COX-inhibitory but absent when the purified and detergent-solubilized enzyme is analyzed.

### COX-inhibitory NIR lowers the mitochondrial membrane potential

**Figure 2:**
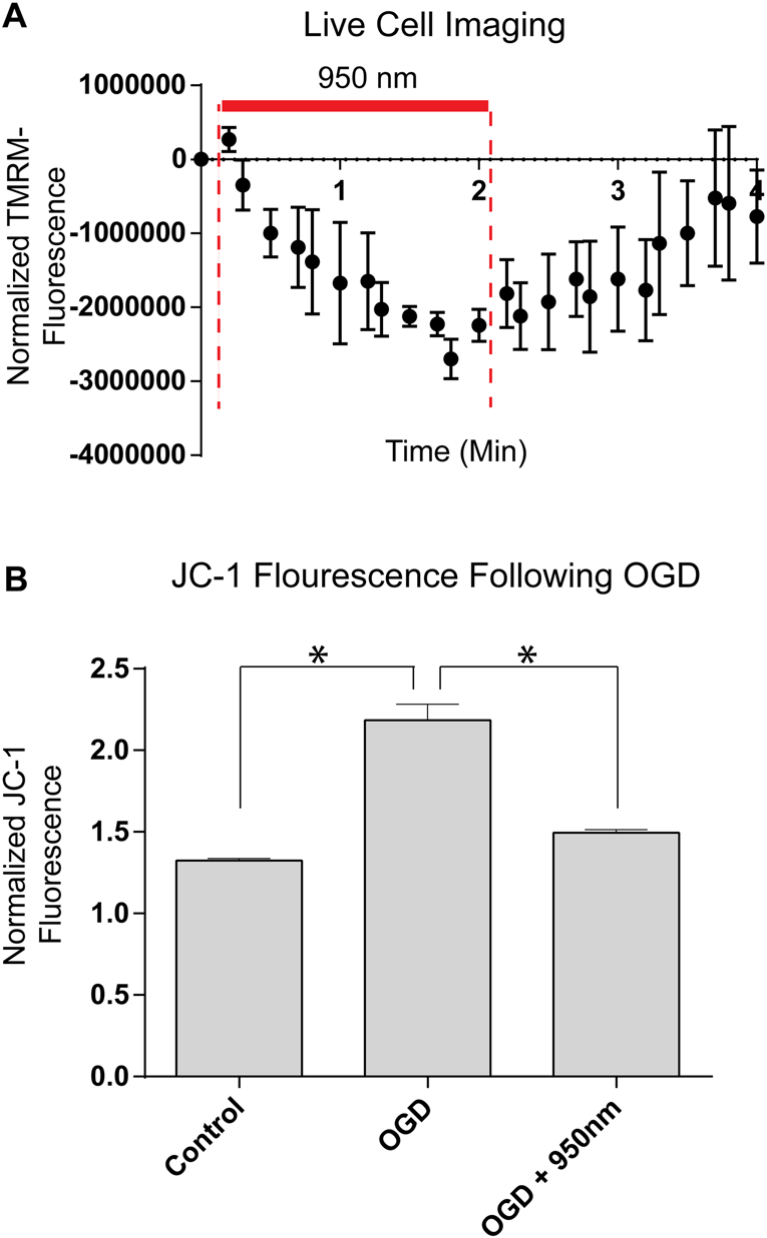
Effect of NIR on mitochondrial membrane potential in cultured HT22 cells. (**A**) Analysis of the mitochondrial membrane potential (ΔΨ_m_) during irradiation with 950 nm NIR in live cells using the probe TMRM (n = 3, time in min). (**B**) Analysis of ΔΨ_m_ during the first 30 min of reoxygenation after 1 h O_2_/glucose deprivation [OGD] imaged in live cells using the probe JC-1. Data are mean values of 96 well live cell fluorescence readings.

Multiple studies have suggested that COX is the rate-limiting step in mitochondrial ETC^23–27^. We therefore hypothesized that COX-inhibitory NIR is protective in conditions of mitochondrial hyperactivity during conditions of stress. Since COX together with ETC complexes I and III generates ΔΨ_m_, a reduction of ETC flux should lead to a reduction of ΔΨ_m_. Therefore, we investigated the effect of NIR treatment in HT22 cells, an immortalized line of cultured hippocampal neurons. These studies utilized fluorescent reporters which precluded analysis with shorter wavelengths (750 nm and 810 nm) that can potentially influence the excitation/emission spectra of red fluorophores. NIR treatment, applied via an LED array chip, transiently reduced ΔΨ_m_ during irradiation in HT22 hippocampal neurons (Fig. 2A, red bar, as shown with 950 nm NIR) and withdrawal of irradiation resulted in a rapid restoration of ΔΨ_m_. Oxygen-glucose deprivation (OGD) resulted in increased ΔΨ_m_ during early reoxygenation (30 min) in HT22 cells; importantly, COX-inhibitory NIR treatment prevented this increase in ΔΨ_m_ (Fig. 2B).

### NIR limits ROS production and cell death *in vitro*

**Figure 3:**
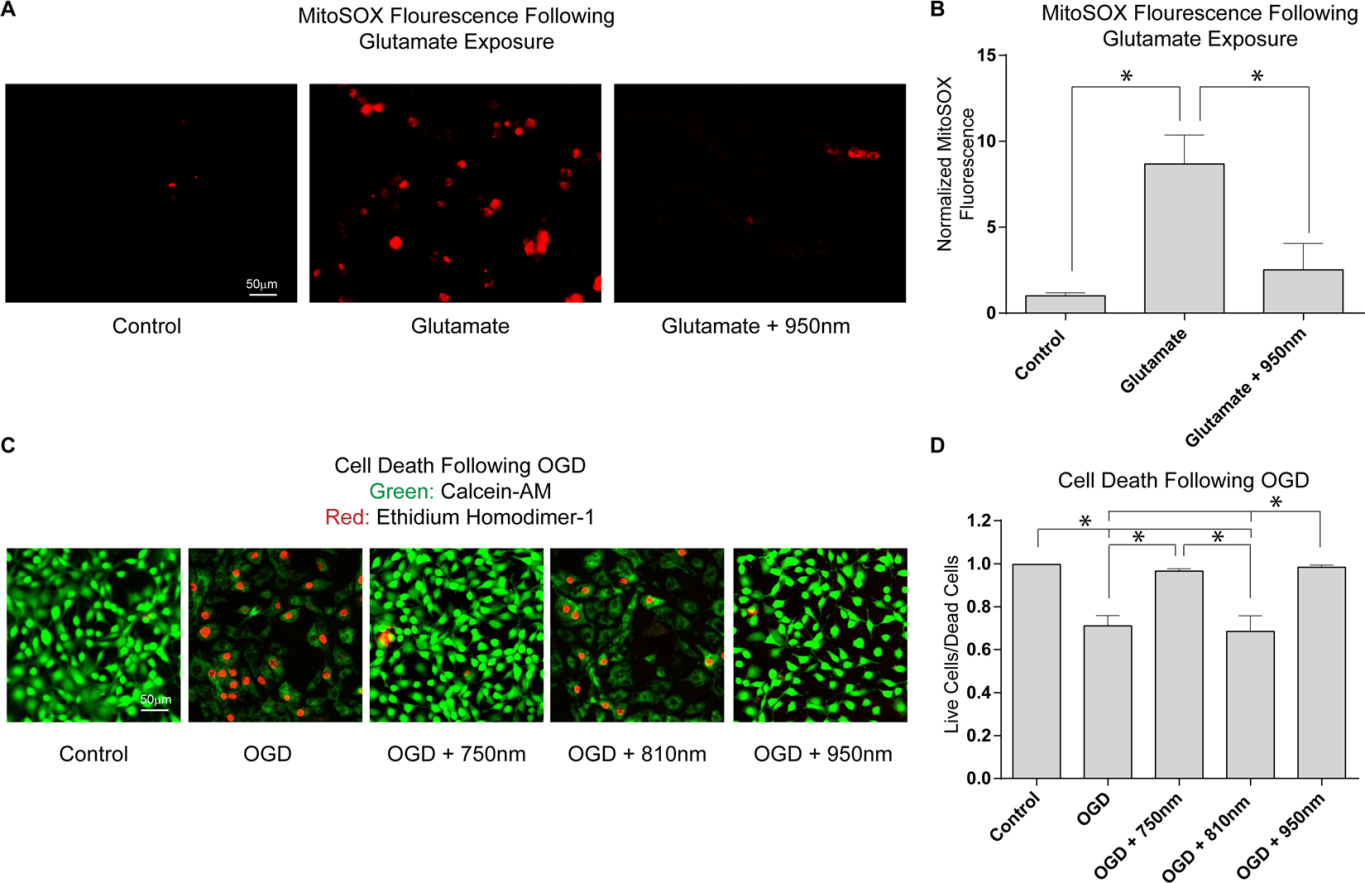
Effect of NIR on cultured HT22 cells following glutamate exposure and OGD. (**A** and **B**) Glutamate induced oxytosis in HT22 cells labeled with MitoSOX (n = 4 per group, *p < 0.05). (**C** and **D**) % Viability of control cells (green live/red dead), cells subjected to simulated I/R (1 h OGD + 24 h reoxygenation), and cells exposed to I/R treated with excitatory NIR (810 nm) and each inhibitory NIR wavelength (750 or 950 nm, n = 6 per group; *p < 0.05).

HT22 cells respond to glutamate exposure with a well-established burst of mitochondrial ROS and cell death through a form of oxidative stress-induced apoptosis termed oxytosis^28–31^. To determine if NIR mediates mitochondrial ROS induced cell death independent of oxygen deprivation, we treated HT22 cells with glutamate with and without NIR treatment. As above, NIR treatment is limited to a longer wavelength (950 nm) to avoid interaction of the treatment wavelength with the excitation/emission spectra of the fluorescent reporter (MitoSOX). Glutamate exposure produced a significant 8-fold increase in MitoSOX fluorescence. NIR irradiation limited this increase in MitoSOX fluorescence in glutamate exposed cells (Fig. 3A and B).

The neuroprotective effect of NIR in HT22 cells was evaluated using a 60-minute OGD exposure followed by reoxygenation. OGD and reoxygenation caused neuronal death in the control cells detected as a loss of calcein-AM (green) fluorescence and an increased number of cells with disrupted plasma membrane detected with ethidium homodimer-1 (red) fluorescence. In contrast, treatment with inhibitory NIR wavelengths (750 nm and 950 nm) rescued neurons from cell death. Importantly, and as predicted by our model, stimulatory NIR (810 nm) provided no protection against OGD and reoxygenation (Fig. 3C and D).

### NIR limits ROS production following global brain ischemia

**Figure 4:**
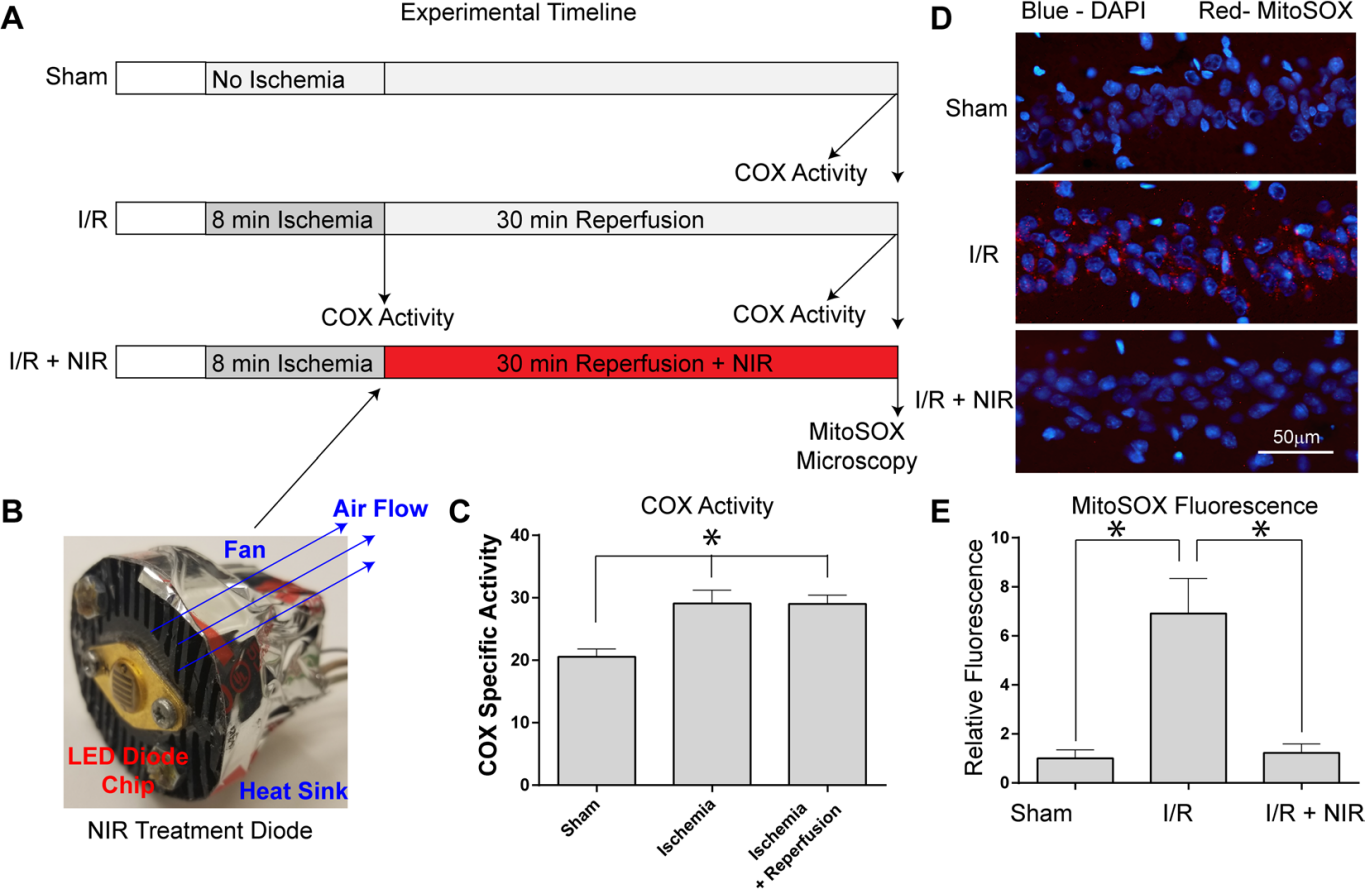
(**A**) Experimental timeline of COX specific activity and MitoSOX experiments. (**B**) LED array 60 chips, used for cell culture and animal studies, were mounted on heat sinks together with a small fan operated in reverse mode, eliminating unspecific heating of the diodes. (**C**) COX activity (defined as consumed O_2_ (μmol)/(min•total protein (mg)), analyzed in brain tissue homogenates after global brain ischemia and ischemia/reperfusion is significantly increased compared to controls (n = 5 per group; *p < 0.05 compared to sham). (**D**) Treatment with NIR limits MitoSOX fluorescence. Nuclei were labeled with DAPI (blue) and mitochondrial ROS were detected with MitoSOX (red). [Top] sham-operated (Sham), [Middle] ischemia followed by 30 min of reperfusion (I/R), [Bottom] I/R plus NIR treatment (I/R + 950 nm). (**E**) Quantitation of red fluorescence shown in D (n = 3 per group; *p < 0.05 compared to sham operated control).

ROS generated during reperfusion is a primary cause of neurologic I/R injury^3^. Reperfusion following ischemia is a stress state where mitochondrial membrane potential (ΔΨ_m_) hyperpolarization occurs and ROS are generated. To demonstrate if COX is hyperactive following ischemia, we harvested brain tissue from control rats, rats that were subjected to 8 min of ischemia, and rats that underwent 8 min of ischemia followed by reperfusion. We measured COX specific activity in the solubilized brain samples and found it to be significantly increased during ischemia and during reperfusion after ischemia (Fig. 4C). We propose that these changes are an underlying cause of ΔΨ_m_ hyperpolarization observed *in vivo* during reperfusion^10^. To test the effect of NIR on reperfusion-mediated ROS generation we analyzed MitoSOX fluorescence, which allows detection of mitochondrial superoxide generation *in vivo*. Eight min of transient global brain ischemia followed by 30 min of reperfusion (Fig. 4A) caused a nearly seven-fold increase in MitoSOX fluorescence and thus ROS accumulation in the CA1 region of the hippocampus (Fig. 4D and E, middle panel, I/R group). Noninvasive transcranial illumination using an LED array chip (Fig. 4B) with COX-inhibitory NIR prevented ROS accumulation, with ROS levels in the NIR treatment group comparable to the sham-surgery control cohort (Fig. 4D and E, compare top panel (sham) with bottom panel (I/R + NIR)).

### NIR treatment is neuroprotective

**Figure 5:**
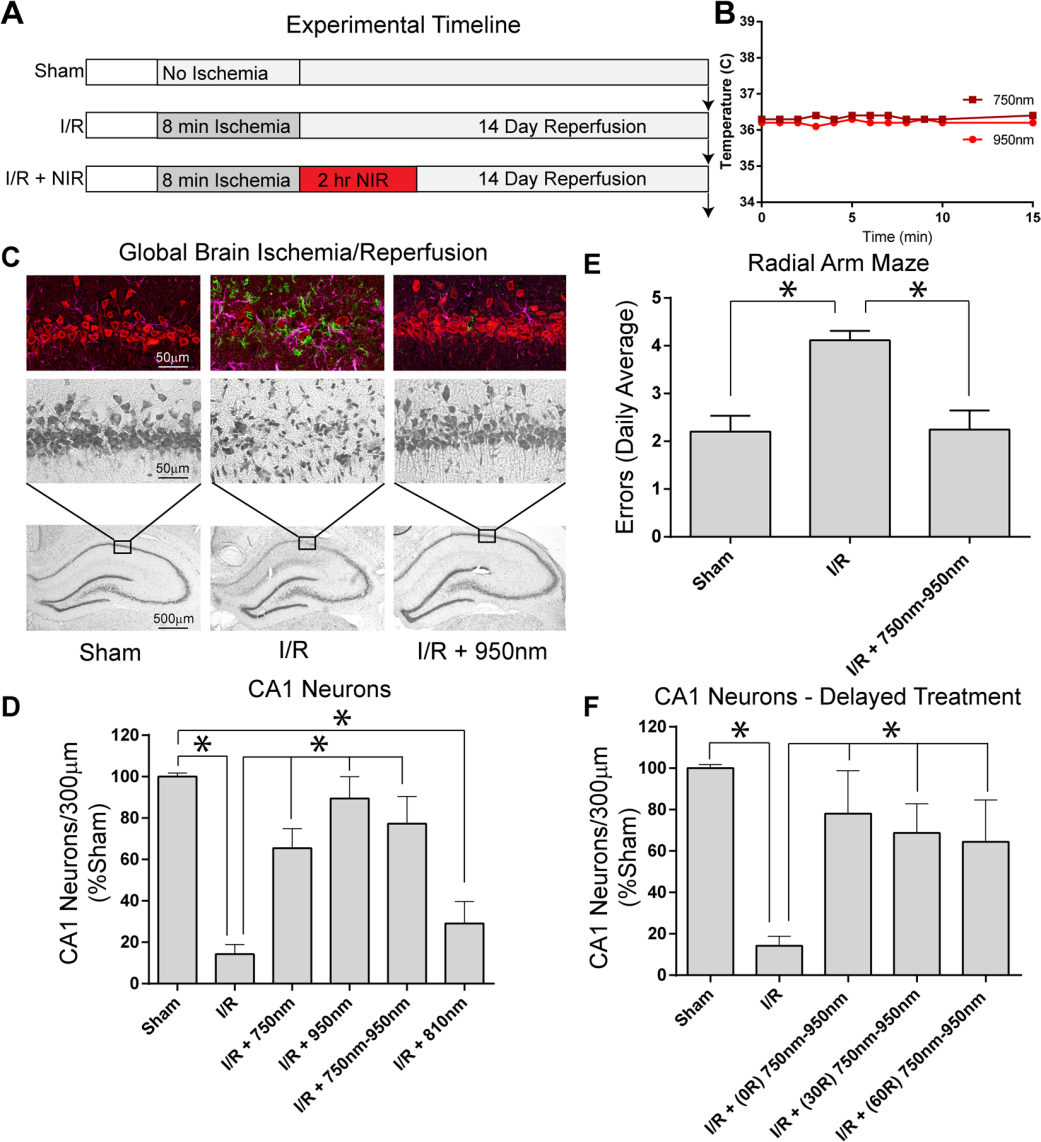
(**A**) Experimental timeline for NIR therapeutic trials. (**B**) Changes in brain temperature during NIR irradiation with LED diode. (**C**) CA1 hippocampal ischemia/reperfusion damage is robustly ameliorated upon NIR treatment during reperfusion. [bottom row] 10× image of Cresyl violet stained hippocampus [middle row] 40× magnification of CA1 hippocampus, [top row] Triple-label immuno-fluorescence for NeuN (red-neuron marker), Iba-1 (green-microglia/macrophage marker), and GFAP (magenta-astrocyte marker). [Left column] Sham-operated animal (Sham), [center column] 8 min ischemia followed by 14 days reperfusion (I/R), [right column] I/R plus NIR treatment (I/R + 950 nm). (**D**) Neuron counts in the CA1 hippocampus (mean ± SEM, n = 8–12 per group; *p < 0.05. (**E**) Radial arm maze performance (n = 10 per group; *p < 0.05 compared to sham). (**F**) Effect of delaying NIR treatment 30 or 60 min after reperfusion (30 R and 60 R; n = 6/group, *p < 0.05 compared to untreated I/R group).

Transient global brain ischemia caused by cardiac arrest results in a neurological injury, the hallmark of which is neuronal cell death and atrophy of the CA1 region of the hippocampus. Animals were randomly enrolled into a treatment-blinded study design, as detailed in Fig. 5A. LED-treatment diodes were coupled with heat sinks and fans (Fig. 4B) to prevent diode heating. These modifications were adequate to prevent brain temperature increase during NIR irradiation (Fig. 5B). Transient global ischemia caused a marked reduction in the CA1 neuron population, shown in Fig. 5C by cresyl violet cell staining and the neuN fluorescent neuronal marker. Gliosis occurs following neurologic damage, characterized by infiltration of inflammatory cells to the site of the injury. I/R injury caused accumulation of microglia and macrophages (Iba-1 immunofluorescent marker) and astrocytes (GFAP) seen in the CA1 by confocal imaging. Treatment with the COX-inhibitory wavelengths (750 nm or 950 nm), or a combination of the two wavelengths, significantly attenuated neuronal loss in the CA1 region. The I/R cohort exhibited an 88% loss of neurons in the CA1 hippocampus at 14 days post-reperfusion, whereas the inhibitory NIR treatment groups showed neuronal loss ranging from only 11% to 35%. As an important control, treatment with stimulatory NIR (810 nm), did not provide neuroprotection in the CA1 hippocampus (Fig. 5D).

### Delayed treatment with NIR

These data demonstrate significant tissue protection when NIR is applied at the onset of reperfusion. It is important to note that clinical application of NIR therapy at the time of resuscitation is feasible for certain applications; however, some implementation barriers may make a delayed treatment more clinically realistic. Therefore, we also evaluated how delaying treatment up to 60 min after reperfusion would affect neuroprotection. We found 78% cell survival when 750 nm-950 nm NIR was applied at reperfusion vs. 64% when applied after 1 h of reperfusion, demonstrating significant neuroprotection with delayed administration compared to untreated controls (Fig. 5F).

### Neurobehavioral improvement with NIR

Ultimately, the goal of preventing I/R injury in the brain is to improve cognitive function following cardiac arrest and resuscitation. Thus, using radial arm maze testing, we examined the effect of NIR on neurobehavioral function using the NIR treatment (750 nm + 950 nm), which is most effective at inhibiting COX, reducing mitochondrial respiration, and provided robust neuroprotection in the histologic studies (Figs. 1C and D, 5C and D). Following I/R, animals had an increase in navigation errors. Improvement of neurobehavioral function after dual wavelength (750 nm + 950 nm) treatment improved performance to the level of the sham-operated cohort (Fig. 5E).

## Discussion

NIR has gained increasing attention as both a diagnostic and therapeutic tool, and has obvious clinical advantages: NIR therapy is (1) non-invasive, (2) immediately effective and reversible, (3) non-pharmaceutical/non-chemical, and (4) proven safe for human treatment. NIR between 700–1000 nm readily permeates mammalian tissue because photon absorption by water and hemoglobin is minimal at these wavelengths^32,33^. Depending on the wavelength and tissue type, NIR penetrates tissue (~2–9 cm)^34,35^ and is increasingly used to probe structure and oxygenation of the brain (e.g., cerebral oximetry is based on penetration of NIR through the skull and into the brain). Photobiomodulation has been tested as a potential approach for multiple therapeutic applications including nerve regeneration, peripheral neuropathy, stroke, and wound healing^12,36–46^. However, some results have been contradictory^34,47–49^ or failed to show clinical efficacy^34^. This may, in part, be explained by different treatment approaches, for example utilizing different energies or duration of the treatment, treatment during different phases of injury progression, or even similar total energies used but applied for different durations can lead to conflicting results^50^. Another limitation is that most studies have focused on a single or very few wavelengths (rather than performing a more comprehensive spectral analysis) when investigating photobiomodulation by NIR. Finally, most studies have shown that NIR exclusively stimulates COX activity and mitochondrial respiration^17,18,51–53^.

In this study, to further our understanding of photobiomodulation, we used a novel approach to uncover specific NIR wavelengths, which directly affect COX activity. We measured oxygen consumption with isolated COX while scanning the spectrum of wavelengths in the near infrared range. We confirm that an established photostimulatory wavelength (810 nm) had the predicted effect of stimulating enzyme activity (Fig. 1C). In addition, and contrary to the common finding that NIR causes COX activation, we report that 750 and 950 nm NIR suppressed oxygen consumption by COX. Moreover, combined irradiation with the inhibitory wavelengths further reduced COX activity. Our findings demonstrate that specific wavelengths can have a distinct impact on COX activity. With our identified inhibitory wavelengths, we have established a new tool for normalizing hyperactive mitochondrial function, such as during reperfusion following ischemia.

COX-inhibitory but not COX-activating NIR provided protection against ischemia/reperfusion injury in neurons and intact brain (Fig. 5). Nonetheless, the precise mechanisms by which tissues benefit from NIR therapy has not been fully characterized. Previous studies reported that NIR could provide neurologic benefit through activation of COX by stimulating controlled release of ROS and subsequent recruitment of antioxidant defenses^54^, or promote ATP synthesis, providing relief from energy depletion during ischemia^55^. NIR was also reported to activate angiogenesis^56^, to increase COX levels^57^, and to modulate protein synthesis^58^ and activity^46^. The latter proposals may be relevant and applicable to later phases of I/R injury, for example to aid in tissue repair and remodeling, but cannot explain the acute consequences of NIR observed in the current study.

**Figure 6:**
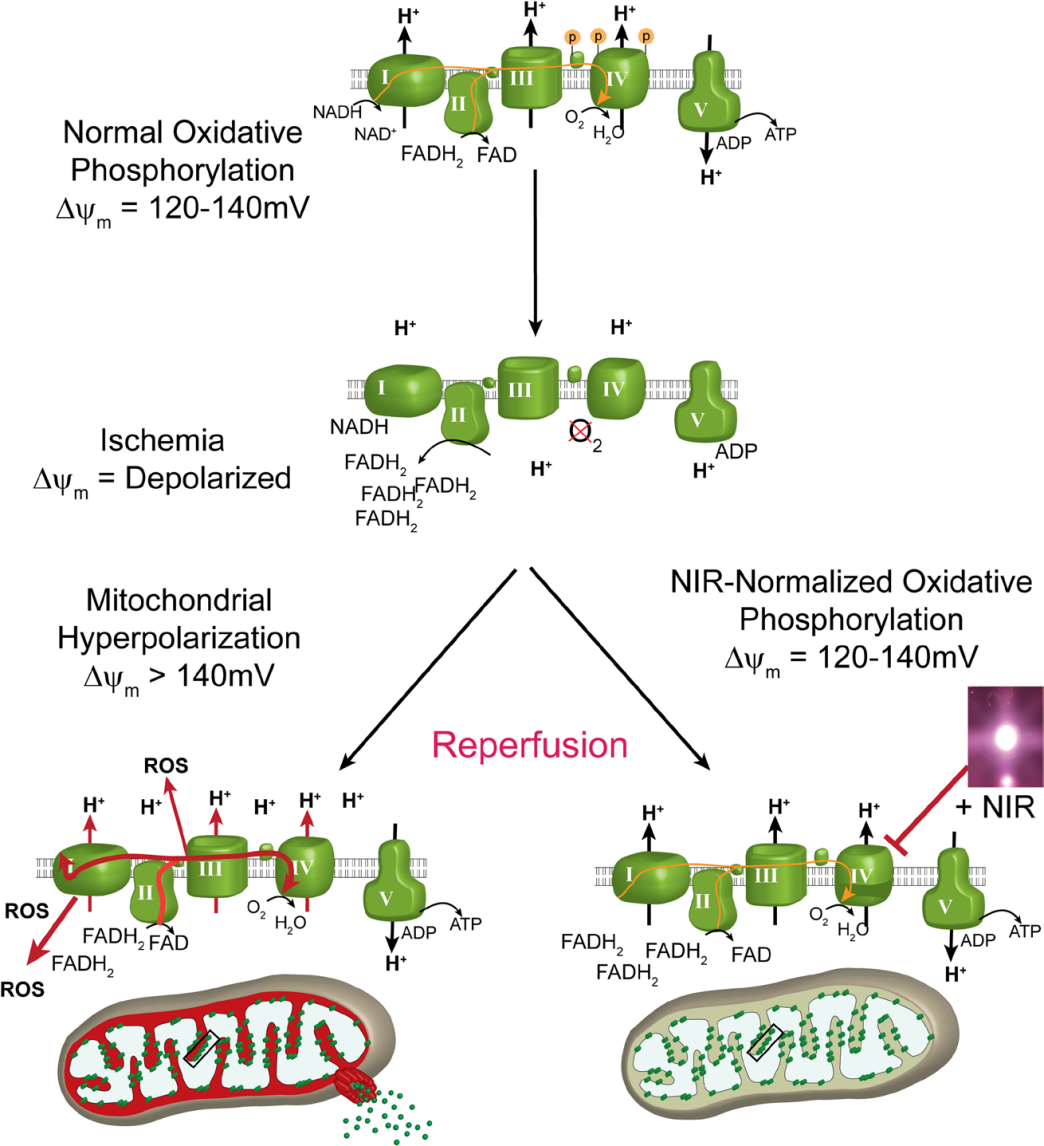
Model of neuroprotection via inhibition of cytochrome *c* oxidase (COX) with infrared light (NIR) during reperfusion. Activity of COX is down-regulated (controlled) via phosphorylation under normal conditions (yellow “P” on COX and cytochrome *c*). During ischemia, COX becomes dephosphorylated but cannot operate due to the lack of O_2_, while NADH and succinate accumulate. At the onset of reperfusion and in the presence of O_2_, the ETC operates at maximal activity, creating pathologically high mitochondrial membrane potentials (ΔΨ_m_), which leads to reverse electron flux, excessive ROS production at ETC complexes I and III, and mitochondrial loss. Transient inhibition of COX with NIR during reperfusion prevents ΔΨ_m_ hyperpolarization, the production of ROS, and thus cell death (adapted from Sanderson *et al*. 2013).

Our data supports a model (Fig. 6), in which inhibitory NIR prevents hyperpolarization of ΔΨ_m_ during reperfusion, thus preventing ROS generation, the primary cause of neuronal death in I/R injury^59,60^. The relationship of ΔΨ_m_ and ROS production is not linear; indeed, when ΔΨ_m_ exceeds 140 mV ROS generation increases exponentially. Consequently, even a relatively small reduction of ΔΨ_m_ through NIR-mediated inhibition of COX would result in a large reduction of ROS. Consistent with our model, we report that treatment of HT22 cells with COX-inhibitory NIR reduces ΔΨ_m_ (Fig. 2A) and that NIR treatment during reperfusion after global brain ischemia prevented accumulation of mitochondrial localized ROS *in vivo*, as seen in untreated hippocampi (Fig. 4). To verify that the clinical benefit was, in fact, a corollary of neuroprotection from NIR treatment, we measured spatial learning and memory function in rat cohorts. Strikingly, dual wavelength treatment, which is highly neuroprotective, improved navigation performance of the radial arm maze to control levels (Fig. 5E).

In conclusion, we report that NIR therapy is an effective treatment for global brain ischemia. NIR treatment provides a safe, non-invasive option for preventing I/R injury in the brain, with clear clinical advantages because it eliminates the production of ROS rather than trying to scavenge ROS already generated, thereby circumventing the inherent limitations of pharmacological treatments of reperfusion injury, which rely on blood flow. We propose that novel photo-suppressive NIR wavelengths reduce neurological injury by inhibiting COX, which prevents ΔΨ_m_ hyperpolarization during reperfusion, thus reducing cytotoxic ROS generation (Fig. 6). We argue that timing of treatment, together with the application of appropriate NIR wavelengths, are crucial for neuroprotection: inhibitory (but not stimulatory) NIR, applied in the early phase of reperfusion, prevents ROS generation and thus tissue injury and cell death signaling in the setting of global cerebral ischemia.

## Materials and Methods

### Chemicals, reagents, and light emitting diodes

Chemicals and reagents were obtained from Sigma-Aldrich (St. Luis, Montana, USA) unless otherwise stated. Light emitting diodes (LEDs) were obtained from Roithner Lasertechnik (Vienna, Austria). For animal experiments LED array 60 chips were used (750 nm, LED750-66-60; 810 nm, LED810-66-60; 950 nm, LED950-66-60). Diodes were mounted on heat sinks (black aluminum, 47 × 20 for LED array 60 chips) together with a small fan (EC3010M05X; Evercool, New Taipei City, Taiwan) operated in reverse mode (see Fig. 2B). Diodes were calibrated with an optical power and energy meter (842-PE; Newport, Irvine, California, USA) and operated with an energy density of 200 mW/cm^2^. For oxygraph experiments, low energy single diodes were used bundled in pairs of three diodes for each wavelength and operated at the manufacturer’s recommended power settings (750 nm, LED750-33AU, operated at 1.85 V and 15 mW power output; 810 nm, LED810-xxAU, operated at 1.7 V and 22 mW power output; 950 nm, LED-950-525, operated at 1.5 V and 32 mW power output) and placed into the quartz windows of the light-protected oxygen electrode chamber.

### Isolation of regulatory competent COX

Mitochondria and, subsequently, COX were isolated from bovine liver as described in detail^61^. In brief, mitochondria were purified by differential centrifugation, solubilized with Triton X-100, and ultra-centrifuged. The supernatants, which contain COX, were subjected to DEAE Sephacel ion exchange chromatography (GE Healthcare, Pittsburgh, PA, USA). The eluted fractions containing COX were supplemented with cholate following ammonium sulfate fractionation. Purified COX was stored frozen at −80 °C until use. Before activity measurements were performed, COX was dialyzed in the presence of ATP and bovine heart cardiolipin to remove cholate bound to nucleotide binding sites on COX, and to replace cardiolipin that was damaged or lost during COX isolation, respectively. COX purity was confirmed by high resolution urea/SDS-PAGE^62^.

### Measurement of COX activity

COX activity was analyzed as described^63^ with the following modifications. Measurements were performed in a final volume of 1 mL in a closed, light-protected oxygen electrode chamber equipped with a micro Clark-type oxygen electrode and closable quartz windows for light application (DW2/2 chamber, Oxygraph system, Hansatech). Experiments were carried out with COX (200 nM) at 20 °C in the presence of 20 mM ascorbate and 1 μM cow heart cytochrome *c* in 50 mM KH_2_PO_4_ (pH 7.4), 1% Tween 20, 10 mM KF, 2 mM EGTA. Oxygen consumption was recorded on a computer and analyzed with the Oxygraph plus software. Turnover number is defined as oxygen consumed (μmol)/(s·COX (μmol)). For the wavelength scan experiments the DW2/2 chamber was integrated into a double beam spectrophotometer (V-570, equipped with an infrared light source; Jasco, Easton, MD, USA) and scans were performed covering the range of 700 to 1000 nm using a scan speed of 20 nm/min and a bandwidth of 8 nm while oxygen consumption rate was recorded. A trend-line was fitted using the moving average function in Microsoft Excel. COX specific activity in brain tissue homogenates was determined as described^63^.

### Mitochondrial respiration measurements

Rat livers were freshly harvested following mitochondria isolation as described above. The effect of NIR on intact mitochondrial respiration was analyzed using the same protocols as described for COX. Mitochondria were diluted to a final concentration of 2 mg/mL in measuring buffer (120 mM KCL, 5 mM KH_2_PO_4_, 3 mM HEPES, 1 mM EGTA, pH 7.2). Following baseline measurements, 10 mM Na-pyruvate and 3 mM malate were added as substrates, oxygen consumption rate (OCR) was recorded for 2 min after which NIR (same energy settings as above) was turned on and applied for 2 min while continuously monitoring OCR. Turnover number is defined as the amount of oxygen consumed (nmol/min per mg protein). OCR after substrate addition was set to 100% and changes during IRL treatment are reported as % change.

### Mitochondrial membrane potential changes

HT22 murine hippocampal neurons were used for all *in vitro* experiments^31^. Cells were plated on glass-bottom culture dishes and experiments were conducted in a microscope chamber on an inverted Axio Observer microscope (Zeiss, Oberkochen, Germany). TMRM (20 µM, Molecular Probes, Waltham, MA, USA) was added to the culture media 30 min prior to imaging. Cells were visualized at 40× magnification and exposures were taken every 30 seconds for the duration of the experiment (approximately 30 min). Cells were irradiated with NIR of 950 nm low energy single diodes (950 nm, LED-950-525, operated at 50 mW power output) for 2 min then NIR was turned off for another 2 min. The z-stacks were set with images collected per 1 µm optical section. Volocity software (Perkin Elmer) was used for image acquisition. Images were evaluated in Volocity software for changes in fluorescence over time, and normalized to control non-irradiated cells. Endpoints with fluorescence measurement (JC-1, TMRM, and MitoSOX) are restricted to one of the 2 identified inhibitory-NIR wavelengths, 950 nm. 750 nm and 810 nm NIR interferes with the excitation/emission spectra for these fluorescent indicators and thus precluded us from testing these wavelengths for parallel effects.

### Glutamate Toxicity in HT22 Neurons

Glutamate toxicity assay was used to interrogate the effect of NIR on ROS generation. HT22 cells were incubated with 5 mM glutamate and 2.5 nM MitoSOX for 8 h as described^31^. Cells were imaged on a Zeiss inverted fluorescence microscope at 20× and at a Z-plane interval of 0.6 μm. For each treatment group, ten spatially distinct and randomly chosen 2-channel (bright-field and fluorescence) images were obtained. Each image was extracted from fifteen sequential micrographs along the Z-axis with extended depth of focus rendered through ZenPro Imaging Software (Carl Zeiss AG). All images were analyzed after subtracting background with ImageJ (NIH). ROS was measured as the integrated density of MitoSOX fluorescence, normalized to the number of cells and averaged for each experiment. Fold-change was calculated by comparing each treatment to the respective group control (NIR-treated or untreated cells without glutamate).

### Oxygen Glucose Deprivation

To model ischemia-like conditions *in vitro*, cells were exposed to transient oxygen and glucose deprivation (OGD)^64,65^. In brief, the culture medium was replaced twice with serum- and glucose-free medium bubbled with 95% N_2_ and 5% CO_2_. The glucose media-deprived cultures were then placed in a Billups-Rothenberg (Del Mar, CA, USA) modular hypoxic chamber, which was flushed for 10 min with 95% N_2_ and 5% CO_2_ and then sealed. The chamber was placed in a water-jacketed incubator at 37 °C for 60 min and then returned to 95% air, 5% CO_2_ and glucose-containing medium for the period of time indicated in each experiment. Control normoglycemic cultures were incubated for the same periods of time at normoxic conditions. Cell death was assessed by incubating cells with Calcein-AM to detect active metabolism (green + no red = live) and Ethidium Homodimer 1 to detect disrupted plasma membrane (red nuclei = dead) (Invitrogen Live/Dead). Random sampling of eight regions of interest were imaged at 20× and live and dead cells were quantified by blinded investigators. Cell death/survival is presented as the ratio of live/dead cells. For evaluation of mitochondrial membrane potential (ΔΨ_m_) changes after OGD, we preincubated a separate cohort with the ratiometric ΔΨ_m_ sensitive dye, JC-1 (2 µg/mL, Molecular Probes, Waltham, MA, USA). Excitation at 488 nm and fluorescence emission at 525 nm and 590 nm were recorded in a fluorescent plate reader (Molecular Devices) and reported as the ratio 590 nm/525 nm intensity.

### Model of global brain ischemia

Animal use protocols were approved by the Wayne State University Institutional Animal Care and Use Committee and all experiments were performed in accordance with the IACUC protocol and relevant regulations. Global brain ischemia and reperfusion was induced in Sprague Dawley rats as previously described by our lab^66–69^, using the method commonly referred to as the 2-vessel occlusion/hypotension model of brain ischemia^66,70^. Briefly, the carotid arteries were isolated through a ventral midline incision and transiently occluded with vascular clips. To aid in reduction of cerebral blood flow through the vertebrobasilar supply, the systemic blood pressure was reduced to achieve a mean arterial pressure of 30 mmHg ± 1 mm Hg by arterial blood withdrawal. Reperfusion was initiated by removal of the carotid clips and re-infusion of withdrawn blood. The model of global brain ischemia produces a well-documented reduction in cerebral blood flow during the ischemic phase. Reperfusion is induced with removal of aneurism clips from the carotid arteries and re-infusion of the withdrawn blood. Our previous studies detail the reproducible loss of cerebral blood flow in this model measured with laser Doppler flow^68^. In addition, standard changes in hemodynamics^68^, brain temperature, blood gasses^71^ and blood glucose^67^ are documented in our previous studies.

### Administration of NIR treatment

NIR was administered by direct illumination through the intact scalp and skull via LEDs placed 1.5 cm from the dorsal scalp. Power density at the scalp surface was 50 mW/cm^2^. This placement was optimized to produce no measurable increase in brain temperature (<0.1 °C, measured with implanted thermocouples, Fig. 5B). The scalp was shaved and a drop of glycerol was applied to the shaved area to aid NIR penetration^72^. Animals were treated with (1) the COX-inhibitory NIR wavelength 750 nm; (2) the COX-inhibitory 950 nm wavelength; (3) combined administration of both inhibitory wavelengths, or, as a control, (4) the COX-excitatory wavelength, 810 nm (n = 7–12 per group). NIR irradiation was initiated at the onset of reperfusion and was continued for 120 min.

### Randomization of Animals to Experimental/Treatment Groups

Experimental group allocation was determined utilizing a blinded/randomized study design. Target group enrollment number was determined by power analysis based on effect size in our previous neuroprotection studies. This protocol was driven by a registrar who is neither one of the surgeons nor the investigator responsible for tissue analysis. The surgeon began the experiment by enrolling a rat and surgically preparing the animal for global brain ischemia. The registrar used a random number generator to determine group allocation of sham-operated control, I/R – untreated, I/R + 750 nm, I/R + 810 nm, I/R + 950 nm, or I/R + 750 nm/950 nm. The registrar initiated treatment with light diodes or dummy diodes in the case of untreated controls. A numbering system is used to identify treatment allocation in the lab record that was known only to the registrar until unblinding after data quantification. The brains were processed and analyzed by a technician provided only the animal number and no group allocation details. Animals that died or were euthanized prior to the predetermined endpoint of the study were not included in the histologic evaluation (sham-operated control: 0/11, I/R – untreated: 8/17, I/R + 750 nm :2/11, I/R + 810 nm: 2/11, I/R + 950 nm: 1/8, or I/R + 750 nm/950 nm: 0/8). Once data acquisition was finalized, the resultant neuron counts were unblinded and organized into treatment groups for statistical analysis.

### Histological Assessment

At 14 days of reperfusion, anesthetized animals were transcardially perfused with PBS, then perfusion-fixed with 4% paraformaldehyde in PBS. The brain was removed, cut into 5 mm-thick coronal blocks, and postfixed in 4% paraformaldehyde in PBS for 2 h. Blocks were cryprotected in 30% sucrose in PBS for 24–48 h. The hippocampal formation was serially sectioned between 2.1 and 3.3-mm posterior to bregma at a thickness of 20 μm in the coronal plane and mounted onto slides, air-dried overnight, and then stained with cresyl violet. From the original set of sections, a total of 3 slides were selected from −2.3, −2.5, and −2.7 posterior to bregma, totaling 9 slides per animal. Digital images were taken of the CA1 hippocampus under light microscopy at 40× magnification. To eliminate counting bias, 300 μm-long sections of CA1 hippocampus were randomly chosen, and neuron counts for each image were performed by 2 investigators blinded to the study group and to each other’s counts. Inter-rater reliability, determined by calculating the correlation coefficient between the ratings of the two observers, was found to be 0.99. Only cells that were morphologically identified as CA1 pyramidal neurons and that had a visible nucleus were counted. Neurons showing clear accumulation of globular materials in the cytoplasm, evidence of nuclear fragmentation, shrunken perikarya, or darkly stained nuclei of reduced size were not counted as viable neurons.

### Immunofluorescence

After 8 min of ischemia followed by 14 days of reperfusion, rat brains were fixed and analyzed by triple-label immunofluorescence as previously described^66^. Primary antibodies for Iba-1 (ab5076, Abcam, Cambridge, MA, USA), GFAP (ab16997, Abcam), and neuronal nuclei (NeuN, MAB377, Millipore, Billerica, MA, USA) were used to identify microglia, astrocytes, and neurons, respectively. Primary antibodies were labeled with Alexa Fluor conjugated secondary antibodies Alexa Fluor −488, −647, and −546. Immunofluoresence images were acquired on a Leica (Wetzlar, Germany) LSM510 confocal microscope, under a 63× oil-immersion objective. A series of 10 optical sections was taken every 0.25 μm in the z-plane, stacked into z-stacks of 2.5 μm, and shown as a z-projection of the total z-stack using ImageJ software (National Institutes of Health, Bethesda, MD, USA).

### *In situ* detection of ROS with MitoSOX

Mitochondrial ROS generation was detected *in vivo* using the fluorescent indicator MitoSOX (Molecular Probes). Rats were pretreated with a single bolus of MitoSOX (100 µg) via intravenous infusion 30 min prior to induction of ischemia, subjected to global brain ischemia for 8 min, and underwent reperfusion with or without NIR treatment. At 30 min post-reflow, anesthetized animals were transcardially perfused with PBS, then perfusion-fixed with 4% paraformaldehyde. The brains were cryosectioned as described above and tissue sections were counterstained with DAPI for nuclear identification and imaged on a Leica SP5 confocal microscope.

### Radial Arm Maze

In a separate cohort of animals subjected to global cerebral ischemia, a radial arm maze (RAM) was used to assess neurologic function. NIR-treatment groups were limited to the best treatment based on the inhibitory effect on COX and histologic studies (950 nm-750 nm). One week following ischemia rats began a mild food restriction to gradually reduce body weight to 80% of the age-adjusted weight prior to the experiment. Two weeks following ischemia rats were placed in an 8-arm Plexiglas maze composed of a central octagonal platform with food rewards placed at the end of each arm of the 8-arm maze (Med Associates, Fairfax, VT, USA). Testing was performed in 2 phases: days 1–7 were the training phase and days 8–14 were considered the testing phase. Errors in the testing phase were recorded when the rat entered an arm where the food reward had already been eaten or entered an un-baited arm. Errors are summated and reported as mean daily error.

### Statistical analysis

Groups were compared using a one-way ANOVA followed by a Tukey’s HSD test for *post hoc* analysis.

## Electronic supplementary material

Supplementary Figure 1
