# Publisher Correction: Inhibitory modulation of cytochrome *c* oxidase activity with specific near-infrared light wavelengths attenuates brain ischemia/reperfusion injury

**DOI:** 10.1038/s41598-018-25184-3

**Published:** 2018-04-25

**Authors:** Thomas H. Sanderson, Joseph M. Wider, Icksoo Lee, Christian A. Reynolds, Jenney Liu, Bradley Lepore, Reneé Tousignant, Melissa J. Bukowski, Hollie Johnston, Alemu Fite, Sarita Raghunayakula, John Kamholz, Lawrence I. Grossman, Karin Przyklenk, Maik Hüttemann

**Affiliations:** 1 Department of Emergency Medicine, Wayne State University School of Medicine, Detroit, MI 48201 USA; 2 Department of Emergency Medicine, University of Michigan Medical School, Ann Arbor, MI 48109 USA; 3 Department of Molecular and Integrative Physiology, University of Michigan Medical School, Ann Arbor, MI 48109 USA; 4 Cardiovascular Research Institute, Wayne State University School of Medicine, Detroit, MI 48201 USA; 5 Department of Physiology, Wayne State University School of Medicine, Detroit, MI 48201 USA; 6 Center for Molecular Medicine and Genetics, Wayne State University School of Medicine, Detroit, MI 48201 USA; 7 College of Medicine, Dankook University, Cheonan-si, Chungcheongnam-do 31116 Republic of Korea

Correction to: *Scientific Reports* 10.1038/s41598-018-21869-x, published online 22 February 2018

In the original version of this Article, Affiliations 1, 2 and 3 were not listed in the correct order. The correct affiliations are listed below:

Affiliation 1:

Department of Emergency Medicine, Wayne State University School of Medicine, Detroit, MI, 48201, USA.

Affiliation 2:

Department of Emergency Medicine, University of Michigan Medical School, Ann Arbor, MI, 48109, USA.

Affiliation 3:

Department of Molecular and Integrative Physiology, University of Michigan Medical School, Ann Arbor, MI, 48109, USA.

This error has now been corrected in the PDF and HTML versions of the Article.

