# Supplementary material for: Inhibitory modulation of cytochrome c oxidase activity with specific near-infrared light wavelengths attenuates brain ischemia/reperfusion injury
Source: Sci Rep. 2018 Feb 22;8:3481. doi: 10.1038/s41598-018-21869-x (PMC5823933; doi:10.1038/s41598-018-21869-x)
Supplement: Supplementary file 1 — Supplementary Figure 1 [file 41598_2018_21869_MOESM1_ESM.docx]

**Inhibitory modulation of cytochrome *c* oxidase activity with specific near-infrared light wavelengths attenuates brain ischemia/reperfusion injury**

Running title: Infrared light attenuates brain reperfusion injury

Thomas H. Sanderson^1,2,3,4,||^, Joseph M. Wider ^2,4,5^, Icksoo Lee^6,7^, Christian A. Reynolds^1,4,5^, Jenney Liu^6^, Bradley Lepore^1^, Reneé Tousignant^1^, Melissa J. Bukowski^1,5^, Hollie Johnston^6^, Alemu Fite^6^, Sarita Raghunayakula^1,2^, John Kamholz^6^, Lawrence I. Grossman^6,4^, Karin Przyklenk^1,4,5^, Maik Hüttemann^6,4,||^.

**
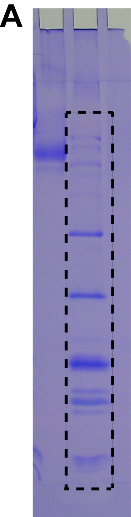
**

**Supplemental Figure 1.** Full length gel from Figure 1A. Dashed lines demarcate crop zone shown in Figure 1A.
